# Glycaemic Response to a Nut-Enriched Diet in Asian Chinese Adults with Normal or High Glycaemia: The Tū Ora RCT

**DOI:** 10.3390/nu16132103

**Published:** 2024-07-01

**Authors:** Ivana R. Sequeira-Bisson, Louise W. Lu, Marta P. Silvestre, Lindsay D. Plank, Nikki Middleditch, Alejandra Acevedo-Fani, Amber Parry-Strong, Kieren G. Hollingsworth, Alexander Tups, Jennifer L. Miles-Chan, Jeremy D. Krebs, Meika Foster, Sally D. Poppitt

**Affiliations:** 1 Human Nutrition Unit, School of Biological Sciences, University of Auckland, Auckland 1024, New Zealand; i.sequeira@auckland.ac.nz (I.R.S.-B.); louise.lu@auckland.ac.nz (L.W.L.); marta.silvestre@nms.unl.pt (M.P.S.); j.miles-chan@auckland.ac.nz (J.L.M.-C.); 2 High Value Nutrition National Science Challenge, Auckland 1023, New Zealand; n.middleditch@massey.ac.nz (N.M.); a.acevedo-fani@massey.ac.nz (A.A.-F.); jeremy.krebs@otago.ac.nz (J.D.K.); meika@edibleresearch.co.nz (M.F.); 3 Riddet Institute, Massey University, Palmerston North 4442, New Zealand; 4 Centro de Investigação em Tecnologias e Serviços de Saúde (CINTESIS), NOVA University of Lisbon, 1169-056 Lisbon, Portugal; 5 Department of Surgery, University of Auckland, Auckland 1023, New Zealand; l.plank@auckland.ac.nz; 6 School of Food and Advanced Technology, Massey University, Palmerston North 4442, New Zealand; 7 Department of Medicine, University of Otago, Dunedin 9054, New Zealand; amber.parry-strong@ccdhb.org.nz; 8 Centre for Endocrine, Diabetes and Obesity Research (CEDOR), Te Whatu Ora, Capital and Coast Health, Wellington 6242, New Zealand; 9 Translational and Clinical Research Institute, Faculty of Medical Science, Newcastle University, Newcastle-upon-Tyne NE1 7RU, UK; 10 Centre for Neuroendocrinology, University of Otago, Dunedin 9054, New Zealand; alexander.tups@otago.ac.nz; 11 Edible Research Ltd., Ohoka, Christchurch 7475, New Zealand; 12 Department of Medicine, University of Auckland, Auckland 1023, New Zealand

**Keywords:** almonds, peanuts, blood glucose, overweight, prediabetes, MRI, ectopic organ fat

## Abstract

Nut-based products are a good source of high-quality plant protein in addition to mono- and polyunsaturated fatty acids, and may aid low-glycaemic dietary strategies important for the prevention of type 2 diabetes (T2D). In particular, they may be advantageous in populations susceptible to dysglycaemia, such as Asian Chinese. The present study aimed to compare effects of a higher-protein nut bar (HP-NB, also higher in total fibre and unsaturated fats, comprising mixed almonds and peanuts) vs. an isoenergetic higher-carbohydrate cereal bar (HC-CB) within the diet of 101 Chinese adults with overweight and normo- or hyperglycaemia. Ectopic pancreas and liver fat were characterised using magnetic resonance imaging and spectroscopy (MRI/S) as a secondary outcome. Participants were randomized to receive HP-NB or HC-CB daily as a 1 MJ light meal or snack replacement, in addition to healthy eating advice. Anthropometry and clinical indicators of T2D risk were assessed fasted and during an oral glucose tolerance test (OGTT), pre- and post-intervention. No significant difference was observed between diet groups for body weight, body mass index, waist or hip circumference, blood pressure, glucoregulatory markers, lipid profile or inflammatory markers over 12 weeks (all, *p* > 0.05). No difference was observed between glycaemic subgroups or those with normal versus high ectopic organ fat. Although HP-NB can attenuate postprandial glycaemia following a meal, no effects were observed for either fasting or glucose-mediated outcomes following longer-term inclusion in the habitual diet of Chinese adults with overweight, including at-risk subgroups.

## 1. Introduction

The prevalence of type 2 diabetes (T2D) has escalated worldwide to 536 million in 2021, and is estimated to rise further to 578 million by 2030 [1]. In China, the total number of adults with T2D has reached 116 million (one in nine adults), the biggest population with T2D globally [1]. Evidence has shown an increased susceptibility to T2D at a low body mass index (BMI) in Asian Chinese compared to many other ethnicities, with a predisposition to pancreatic β-cell dysfunction and insulin resistance, and thus dysglycaemia [2]. It is hypothesized that higher ectopic fat deposition in organs such as the pancreas and liver may typify Chinese individuals who in turn are characterized by the thin-on-the-outside-fat-on-the-inside (TOFI) phenotype [3,4,5]. The TOFI phenotype is an important determinant of hepatic insulin resistance, propagating a negative cycle leading to worsening β-cell dysfunction and T2D [6,7,8]. When exposed to repeated acute postprandial hyperglycaemia, Asian cohorts are subjected to a higher risk of multiple metabolic disorders [9,10], with evidence of adverse glycaemic excursions [11,12,13]. 

Nuts are proposed to play a role in ameliorating the risk of T2D [14], owing to macro-and micro-nutrient content and bioactive compounds that may work synergistically to lower postprandial glycaemia. Whilst not meeting the botanical definition of a ‘true nut’, peanuts (*Arachis hypogaea*, ground nut, legume) and almonds (*Prunus dulcis*, tree nut, drupe) are considered to be within the wider grouping of edible nuts. As such, nuts are a good source of high-quality plant protein, mono- (MUFAs) and polyunsaturated fatty acids (PUFAs), and low glycaemic index (GI) dietary fibre, which are proposed to improve blood lipid profiles, decrease insulin resistance, decrease inflammation and oxidative stress, and modulate endothelial function [15,16,17], as well as potentially promote satiety [18]. There is inconsistent evidence for improvements in blood pressure [17]. Other bioactive compounds present in nuts, such as polyphenol quercetin, may exhibit protective mechanisms including acute inhibition of postprandial α-amylase and α-glucosidase activity and longer-term changes in the gut microbiome [14,19,20]. Lowering the GI or glycaemic load (GL) can benefit postprandial blood glucose and, in turn, may decrease long-term risk of T2D and CVD [10,21]. The beneficial effects of a diet rich in nuts on long-term glycaemic control has been demonstrated in the large European cardiovascular disease (CVD) prevention study PREDIMED (Prevención con Dieta Mediterránea) [22], an intervention in ~7500 participants. Notably, other meta-analyses have shown the Mediterranean diet to be associated with prevention/improvement in fatty liver [23].

Further, a systematic review and meta-analysis of randomized controlled trials (RCTs) in 2019 reported that tree nut and/or peanut consumption had a favourable effect on fasting insulin and insulin resistance (IR) as defined by the homeostatic model assessment model HOMA-IR, although no significant improvements were observed in fasting plasma glucose (FPG) or the longer-term biomarker glycated haemoglobin (HbA_1c_) [24]. The authors proposed that protective effects may be driven through improved insulin sensitivity [24]. 

Compared to the Mediterranean diet, nuts may be a less common food item in the traditional Chinese diet. The Chinese Centre for Disease Control and Prevention reported daily nut consumption to be as low as 3.8 g/day, based on self-reported intake assessed at 150 sites across 31 provinces in the China National Nutrition and Health Survey [25]. This is significantly lower than, for example, the 28 g/day recommended intake proposed in a previous meta-analysis and systematic review of RCTs of walnut interventions [26] and supported in several meta-analyses of prospective cohort studies of nut consumption [27,28]. Certainly, low nut intake has been one of several dietary factors associated with increased CVD risk in the Chinese population [29]. RCTs of nut consumption in Chinese individuals at increased risk of T2D are lacking in the literature. In a Tū Ora postprandial study, we recently reported in a cohort of individuals of Asian Chinese descent that a nut-based snack product significantly improved acute glucose response compared with a typical energy-matched cereal-based snack [30]. This was observed when consumed either alone or with other foods containing a high content of refined carbohydrate (CHO, e.g., white bread), in a group of participants with overweight and prediabetes [30]. A similar acute postprandial study has also recently shown an almond ‘snack’ to suppress both glycaemia and energy intake when compared to an isoenergetic CHO-rich snack in healthy adults both lean and overweight [31]. In the absence of body weight gain when extended over 12 months, this again promoted inclusion of almonds in the diet [32]. Of note in our original study [30], magnetic resonance (MR) assessment of pancreas and liver fat revealed greater suppression of (adverse) postprandial blood glucose peaks by the nut product in the subgroup with low organ fat content, confirming the role of ectopic fat deposition in nutrient response. Fatty pancreas and/or liver worsened this protective dietary response. However, longer-term effects of including a nut-enriched vs. a cereal-rich product in individuals with overweight, in whom blood glucose and ectopic fat are characterized, has not as yet been assessed. Our current Tū Ora 12-week RCT therefore aimed to investigate the effect of a higher-protein, lower-GI nut-enriched product on fasting glycaemia, insulin and related metabolic markers when consumed daily for a duration of 3 months as a replacement for one meal or snack in Chinese men and women with overweight.

## 2. Materials and Methods

A two-arm, parallel-group, dual-centre randomized clinical trial (RCT) was conducted at the Human Nutrition Unit (HNU), University of Auckland, and the Centre of Endocrinology and Diabetes and Obesity Research (CEDOR), Capital and Coast Health, Wellington, New Zealand. Ethical approval was obtained from the National Health and Disabilities Ethics Committee (HDEC), Auckland, New Zealand (18/NTB/1/). The study was registered with the Australian New Zealand Clinical Trials Registry (ACTRN12618000476235). All participants received an information sheet detailing the study protocol and provided written informed consent.

### 2.1. Participants

Participants were recruited from the greater Auckland (AKL) and Wellington (WLG) regions through local and social media advertisements. Eligibility criteria were ethnic Chinese descent defined by self-identification of the participant and both parents, 25–70 years of age, BMI 23–40 kg/m^2^, Finnish Diabetes Risk Score (FINDRISC) ≥ 12 [33], with prediabetes based on the American Diabetes Association (ADA)-defined impaired fasting glucose (IFG), 5.6–6.9 mmol/L [34], and otherwise healthy. Impaired glucose tolerance was not used as an inclusion criterion at screening. Participants were excluded based on change in bodyweight of >5% over the previous 3 months, prior or current significant disease including T2D, current medication likely to affect body weight or glucose metabolism, and tree nut, peanut or other associated food allergies.

All participants attended a screening visit in clinic where body weight, height and blood pressure were recorded and a fasted venous blood sample was collected for the same-day analysis of blood glucose. A total of 187 individuals were assessed for eligibility, of which 106 (AKL n = 90; WLG n = 16) participants were enrolled into the study. All enrolled participants were confirmed with raised FPG within the ADA cut-points at the screen visit. There was then a delay of up to 6 weeks between screening/enrolment and start of the diet intervention with participants grouped into cohorts of n = 10 to facilitate conduct of the trial. 

### 2.2. Diet Groups

All enrolled participants were randomized to one of two diet groups: (1) higher-protein nut bar (HP-NB) or (2) isoenergetic higher-CHO cereal bar (HC-CB). A computer-generated random number sequence provided randomization tables stratified for the two clinical sites. Both sites were given the same randomization sequence (i.e., an equal ratio of two diet groups: 1:1). No block randomization was used. The trial was unblinded, with open allocation to diet group. Ingredient composition of HP-NB and HC-CB products has been detailed previously [30]. Briefly, the HP-NB was formulated to contain (i) the recommended daily serving of nuts > 28 g/day [26] comprising almonds and peanuts, (ii) higher total protein, (iii) higher total and unsaturated fat and (iv) lower total CHO and free sugars. The HC-CB was formulated to comprise (i) matched energy content, (ii) higher cereal content, (iii) higher total CHO and free sugars and (iv) lower total fat (Table 1).

Both products were manufactured by the High Value Nutrition Science of Food team based at the Riddet Institute, Massey University FoodPILOT (Palmerston North, New Zealand) with manufacturing advice provided by The Griffin’s Food Company™ (Auckland, New Zealand) and the NUKU ki te Puku^TM^ (Wellington, New Zealand) coalition of Māori food and beverage businesses. 

### 2.3. Study Design

**Figure 1:**
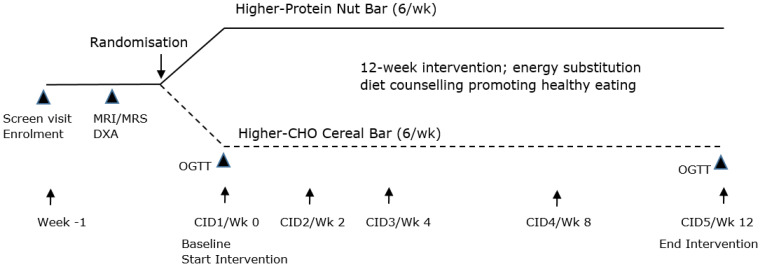
Study design. CHO, carbohydrate; CID, clinical investigation day; Wk, week; OGTT, oral glucose tolerance test; MRI, magnetic resonance imaging; MRS, magnetic resonance spectroscopy; and DXA, dual-energy X-ray absorptiometry.

Each participant attended 5 clinical investigation days (CIDs), as shown in Figure 1. CID1/baseline (week 0), CID 2 (week 2), CID 3 (week 4), CID 4 (week 8) and CID 5/end of study (week 12). All CIDs were completed in the morning, with participants fasted overnight prior to each clinic visit. Following the start of the diet intervention, participants were asked to maintain their current dietary, medication and lifestyle habits over the 12-week study period. A week prior to CID1, each participant underwent dual-energy X-ray absorptiometry (DXA) scanning to assess total body and abdominal fat, and magnetic resonance imaging (MRI) and spectroscopy (MRS) to assess abdominal, pancreatic and liver fat.

At CID1 and CID 5, assessments comprised anthropometry, blood pressure and fasted blood tests including FPG, insulin, HbA_1c_, lipid profile and inflammatory markers. Additionally a 2 h oral glucose tolerance test (OGTT, 75 g glucose) was conducted, with glucose and insulin sequentially assessed at 0, 30, 60, 90 and 120 min. All blood samples were stored frozen at −80 °C until batch analysis of all participants upon completion of the intervention.

All participants completed a 10-question Eating Habit Questionnaire (EHQ) at baseline to record habitual dietary pattern and meal or snack times [36]. Based on this information, a dietitian instructed participants to incorporate HP-NB or HC-CB test products into their daily diet, substituting for one meal or snack at least 5 days per week. Participants were provided with these products at each CID visit, with a total of 72 bars dispensed during the 12-week intervention (6/week). The dietary plan was energy substitution, not addition, across the intervention period. Dietary counselling on healthy eating habits to decrease risk of T2D was provided, based on the Eating and Activity Guidelines for New Zealand Adults, Chinese Dietary Guidelines [37] and China Medical Nutrition Therapy (MNT) Guidelines for Diabetes [38]. These recommendations focused on the 4 macronutrient groups. In summary, recommendations were to avoid added sugars; increase fibre intake from whole grains, legumes, and a variety of colourful fruits and vegetables; decrease saturated fat from animal products and increase unsaturated fat from plant-based products; increase lean meat and meat alternatives; moderate alcohol consumption; and limit salt intake. Participants were requested to not begin vitamin or mineral supplements during the study. 

At CID2, CID3 and CID4, body weight was recorded and fasted blood collected for assessment of FPG and insulin.

### 2.4. Anthropometry

Body weight and height were assessed using a calibrated scale (AKL: Mettler Toledo Spider, Colombus, OH, US; WLG: Tanita Corporation, Tokyo, Japan) and stadiometer (AKL: Seca 222, Hamburg, Germany; WLG: Holtain Ltd., Crymych, UK), while lightly clad and without shoes or a hat. Waist and hip circumference were measured using a nonstretch anthropometric tape (Abbott Laboratories, Green Oaks, IL, USA). Blood pressure was measured seated following >3 min rest, on the non-dominant arm using an automated Digital Critikon Dinamap Sphygmomanometer (GE Healthcare, Shanghai, China).

### 2.5. Body Composition—DXA

DXA whole-body scans were conducted in the body composition laboratory of the Department of Surgery, University of Auckland (iDXA, software v.15, GE-Lunar, Madison, WI, US), and CEDOR, University of Otago, Wellington (Horizon DXA system, Hologic, Marlborough, MA, USA) using standardised imaging and body positioning protocols [39]. Total fat mass (TFM), total fat-free mass (FFM), TFM percentage (%), abdominal fat mass (AbFM) and abdominal fat mass percentage (AbFM%) were measured in the supine position as previously described [30].

### 2.6. Body Composition—MRI and MRS Organ Fat Imaging

MRI/MRS imaging was conducted on a 3T Magnetom Skyra scanner (Siemens, Munich, Germany) in both AKL and WLG, located at the Centre for Advanced Magnetic Resonance Imaging (CAMRI), University of Auckland, and Pacific Radiology, Wellington, respectively. Fast sagittal localizing abdominal images from the diaphragm to pelvis were acquired using the 3D dual gradient-echo sequence (VIBE) 2-point Dixon method [40]. Visceral (VAT) and subcutaneous adipose tissue (SAT) were quantified from a single fat fraction map at the L4-L5 intervertebral disc space [41] using ImageJ v1.51 [42]. Pancreas fat was determined using the ‘MR-opsy’ method [43] with thresholding (1–20%) applied to eliminate any inclusion of non-parenchymal tissue. MRS was performed using a respiratory-gated sequence [44], and liver fat was calculated using the SIVIC software v0.9.105 [45] from the area under the curve (AUC) of water and fat peaks from non-water-suppressed spectra and presented as percentage volume/volume. Pancreas images from 3 participants contained artefacts and could not be analysed; hence, pancreas fat percentage (%) was measured in 98 participants. Additionally, the spectroscopy signal obtained from one participant could not be analysed; hence, liver fat percentage (%) was measured in 100 participants. 

Based on weighted means as reported in a recent meta-analysis, ≥4.5% was used to classify those with high pancreas fat [8], which aligned with median value of 4.53% calculated from a prior data set from our laboratory [46], and ≥5.6% for those with elevated liver fat [47]. Following quantification and classification based on these internal cut-points, participants with either high pancreas fat and/or high liver fat were characterized as high ectopic fat (HEF). Participants without high pancreas and without high liver fat were characterized as normal ectopic fat (NEF). 

### 2.7. Blood Sample Analyses

At screening, fasting plasma glucose (FPG) was measured in AKL from whole blood using a Reflotron Plus Desk Top Analyzer (Roche, Basel, Switzerland) and in WLG from plasma using a COBAS c331 auto analyzer (Roche, Basel, CH). During the intervention, serum and plasma samples were collected and centrifuged at 1400× *g* for 10 min at 4 °C. Aliquots were then prepared and stored at −80 °C for batch analysis of all participants at the end of the study. Plasma glucose was collected in fluoride oxalate vacutainer tubes and determined using a COBAS c311 hexokinase method auto analyzer (Roche, Basel, CH, Switherland). Serum insulin was collected in a serum-separating tube and determined by electrochemiluminescence immunoassay (ECLIA) using a COBAS e400 auto analyzer (Roche, Basel, CH, Switzerland). Insulin sensitivity was calculated using the Homeostatic Model Assessment of Insulin Resistance (HOMA-IR) method: HOMA-IR = fasting insulin (µU/mL) × FPG (mmol/L)/22.5 [48]. Total cholesterol (TC), HDL-cholesterol (HDL-C) and triglyceride (TG) were measured using a COBAS c311auto analyzer (Roche, Basel, CH, Switzerland). LDL-cholesterol (LDL-C) was calculated using the Friedewald equation [49]. Plasma and neuro-inflammatory markers interleukin-6 (IL-6), high-sensitivity C-reactive protein (hsCRP), Dickkopf-1 (DKK-1), brain-derived neurotrophic factor (BDNF) and S100 calcium-binding protein beta (S100beta; Mybiosource, San Diego, CA, USA) were determined by colorimetry using Human ELISA kits (Abcam, Cambridge, UK).

### 2.8. Compliance Assessment

Daily energy intake (DEI) was self-reported on 3 occasions at CID1/baseline, CID4 and CID5 using a 110-item food frequency questionnaire (FFQ), modified from the 137-item 2008/09 New Zealand Adult Nutrition Survey [50]. The total number of intervention products consumed was also recorded at each CID visit, based on participant home records. Compliance to dietary products over the 12-week intervention was calculated as: Compliance (%) = (total no. bars consumed/total no. bars dispensed) × 100. 

### 2.9. Statistical Analysis

Participant characteristics at CID1/baseline are expressed as mean and standard deviation (SD). An independent sample *t*-test was used to compare between HP-NB and HC-CB diet groups, subgroups with normoglycaemia and prediabetes, and NEF and HEF subgroups. Efficacy endpoints are expressed as mean and standard error of the mean (SEM). Diet group effects at CID5/12 weeks were analysed using two-way repeated measures ANOVA (diet*time interaction) and Bonferroni’s post hoc analysis. Effects of diet at 12 weeks between subgroups with normoglycaemia and prediabetes, and between NEF and HEF subgroups, were analysed using a three-way repeated measures ANOVA, and Tukey’s post hoc multiple comparisons test. At CID1 and CID5, the OGTT incremental area under the curve (iAUC) of glucose and insulin was calculated using the trapezoid method, including both positive and negative peaks. Peak concentration (C_max,_) for glucose and insulin was also recorded from the 2 h curves. Change over 12 weeks for OGTT parameters was compared between HP-NB and HC-CB diet groups using a three-way ANCOVA and Bonferroni’s post hoc analysis. % compliance to treatment was compared between diet groups using an independent sample *t*-test. All data were analysed using Prism 8 (GraphPad Software v8.0, San Diego, CA, USA) in n = 101 and n = 97 participants at CID1 and CID5, respectively. Missing data in repeat measures analyses (anthropometry, clinical samples) were imputed at CID 1/baseline using the group mean and at later time points using the last value carried forward (LVCF) method. Missing data for outcomes assessed only at, or prior to, CID1/baseline (DXA and MRI/MRS body composition) were not imputed. *p* ≤ 0.05 represented statistical significance. 

## 3. Results

### 3.1. Participant Flow and Characteristics at CID1/Baseline

**Figure 2:**
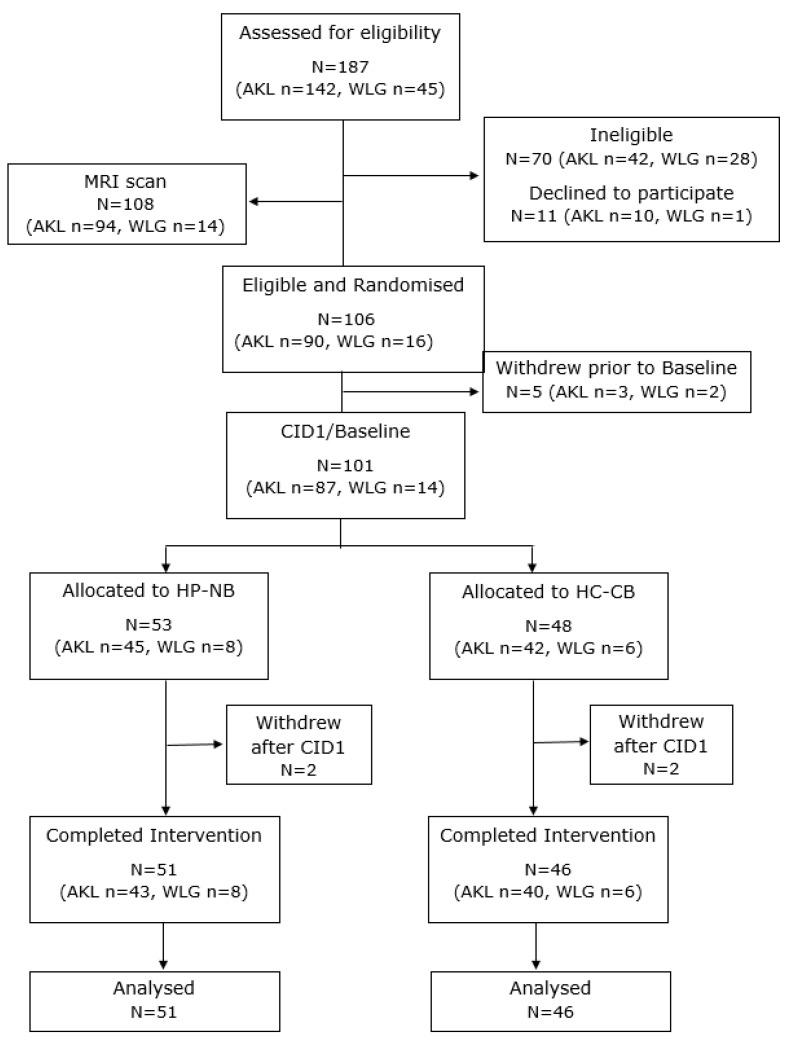
CONSORT flowchart of participants. AKL: Auckland site; CID1: clinical investigation day 1/baseline; HC-CB: higher-CHO cereal bar; HP-NB: higher-protein nut bar; and WLG: Wellington site.

The participant flow chart is shown in Figure 2. A total of 106 participants met the inclusion criteria and were randomized to the two diet groups. Mean (SD) FPG at randomization was 6.1 (0.4) mmol/L. Five participants withdrew prior to CID1/baseline (AKL, n = 3; WLG, n = 2), all from the HC-CB diet group. Hence, 101 participants (M:F, 54:47) were assessed at CID1 and started the intervention, of which 97 then completed the 12-week period. Data analysis was undertaken for the n = 97 completers. At CID1, the mean FPG for all participants was 5.5 (0.6) mmol/L. The baseline characteristics of participants in the HP-NB and HC-CB diet groups, and also glycaemic and ectopic fat subgroups, are shown in Table 2.

### 3.2. Compliance to Treatment

Both diet groups reported high compliance to the test bars over the 12-week intervention. In the 97 completers, the mean compliance was 82% and 85% in the HP-NB and HC-CB groups, respectively, with no difference between groups (*p* > 0.05, Table 3). Over half of all participants (HP-NB: 59%; HC-CB: 67%) consumed the minimum of five (out of six) bars per week across each of the 12 weeks of the study.

### 3.3. Diet Subgroups: HP-NB vs. HC-CB 

There was no significant difference at baseline between the HP-NB and HC-CB diet groups for any parameters other than diastolic blood pressure (DBP), LDL-C and MRI-assessed pancreas fat % (all, *p* < 0.05), which were higher in the HP-NB group by chance. Importantly, age, body weight, and BMI, DXA body composition parameters, and MRI-VAT:SAT ratio and MRS-liver fat% were not significantly different between diet groups. There was also no significant difference in mean FFQ self-reported energy intake (HP-NB: 9.4 ± 0.7 MJ/day; HC-CB: 9.3 ± 0.5 MJ/day) at baseline. Throughout the 12-week intervention, in accordance with the recommendation that product bars be consumed as an energy substitution rather than addition, there was no significant increase in body weight, BMI, waist or hip circumference in either group (time, *p* > 0.05, Table 4), nor was there a difference between the two diet groups (diet*time, *p* > 0.05). FFQ self-reported daily energy intake was also not significantly different between the HP-NB and HC-CB groups (*p* > 0.05). Contrary to our hypothesis, however, there was also no significant difference in change in primary endpoint FPG between HP-NB and HC-CB over 12 weeks (diet*time, *p* = 0.991, see Table 4). Sensitivity analyses comparing imputed and observed ‘raw’ data confirmed no difference in outcomes. There were also no significant diet group differences in change over 12 weeks in fasting insulin, HOMA-IR, HbA_1c_ and lipids, nor any measured inflammatory markers (diet*time, all *p* > 0.05). Unexpectedly, HbA_1c_ increased in both diet groups by ~10% over 12 weeks (time, *p* = 0.016). Conversely, hsCRP decreased in both groups (time, *p* = 0.014). Assessed through OGTT, there was also no significant difference in change over 12 weeks between HP-NB and HC-CB for glucose and insulin ∆C_max_, iAUC_glucose 0–120min_ or iAUC_insulin 0–120min_ response (diet*time, all *p* > 0.05). 

### 3.4. Glycaemia Subgroups: Normal vs. Impaired Fasting Glucose

The mean (SD) FPG at CID1 was 5.5 (0.6) mmol/L; hence, a sub-analysis was conducted to determine responses to the dietary intervention between normo- and impaired fasting glucose subgroups. At baseline, there was no significant difference between the two subgroups in age, body weight or BMI (see Table 2, all, *p* > 0.05). Waist circumference and the majority of metabolic endpoints were significantly higher in the group with prediabetes, including FPG, insulin, HbA_1c,_ HOMA-IR, total and LDL-C, and TG (all, *p* < 0.01). Also significantly higher at baseline in the pre-diabetic group were the MRI-VAT:SAT ratio (*p* = 0.002) and MRI-pancreas fat % (*p* < 0.05). MRS-liver fat % was numerically higher (+2%) but due to high variance did not reach statistical significance between glycaemic subgroups (*p* = 0.089; see Table 2). Over the 12-week intervention, baseline differences between glycaemic subgroups were maintained for multiple endpoints, including many anthropometric, fasting and OGTT glycaemic parameters, lipids and inflammatory markers (all *p* < 0.001). Conversely and notably, however, there was no significant difference in trajectory between diet groups for change in primary variable FPG over 12 weeks when glycaemic status was included in the model (glycaemia*diet*time, *p* > 0.05).

### 3.5. Ectopic Fat Subgroups: Normal vs. High Pancreas and/or Liver Fat

A secondary sub-analysis was also conducted to determine whether there was a differential response to the intervention between the NEF and HEF subgroups. At baseline, HEF participants were of similar age but with significantly higher body weight, BMI, waist and hip circumference (all, *p* < 0.05, see Table 2). There was no difference at baseline in primary endpoint FPG (*p* = 0.206), whilst fasting insulin and HOMA-IR were both significantly higher in the HEF subgroup (*p* < 0.05). DXA-assessed body composition showed significantly higher total FM, FFM and AbFM% (all, *p* < 0.05) in the HEF subgroup, in addition to significantly higher MRI-VAT:SAT ratio, pancreas and liver fat % (all, *p* < 0.001). Over the 12-week intervention, baseline differences between subgroups were maintained for multiple endpoints, including many anthropometric, fasting and OGTT glycaemic parameters (all *p* < 0.001). Conversely and notably, however, there was no significant difference between diet groups for change in primary variable FPG over 12 weeks when ectopic fat status was included in the analysis (ectopic fat*diet*time, *p* > 0.05), or for other anthropometric, fasting and OGTT glycaemic parameters, fasting lipids and inflammatory markers (ectopic fat*diet*time, all *p* > 0.05). 

## 4. Discussion

Our current study found that daily consumption of HP-NB, containing ~28 g of mixed almonds and peanuts, over 12 weeks did not alter markers of glycaemia or insulin resistance relative to an isoenergetic higher-CHO cereal-based bar. There was no evidence that FPG, OGTT 2 h glycaemic response or other T2D-associated blood markers including insulin response improved in this cohort of Chinese adults with overweight. Nor was there an improvement in lipids or inflammatory markers. Based on prior observational studies [27,28] and RCTs [24,52,53,54], we hypothesized that daily consumption of the lower-GI nut-rich HP-NB, when replacing a light meal or snack, would improve some markers of diabetes risk. Previous evidence from our laboratory of glycaemia-related improvements in an acute postprandial setting by the HP-NB intervention [30] was therefore not replicated in our current, longer-term, investigation of fasting and OGTT biomarkers. 

Tree nuts and peanuts have been proposed as an attractive snack food for individuals with dysglycaemia, owing to their pleasant taste and multiple ways of being included in the daily diet. They provide a good source of dietary fibre (~3–13 g per serving), plant-based unsaturated fatty acids (~44–76 en%, mostly MUFA and PUFA), plant-based protein, micronutrients (e.g., vitamins, calcium, magnesium, potassium, etc.) and polyphenols (e.g., α-Tocopherol) [55] while being low in saturated fatty acids (~4–16 en%) and available CHO (~12–30 en%), making them a potential candidate for attenuating the GI response to a meal, and potentially improving longer-term glycaemic control [21,56]. A systematic review and meta-analysis of five prospective cohort studies reported that weekly consumption of four servings of ~28 g of tree nuts, peanuts and legumes reduced the relative risk of T2D by 13% [27]. A later systematic review and meta-analysis of four prospective studies reported a 39% reduction in mortality from T2D per 28 g of nuts and peanuts consumed/day [28]. In addition, a meta-analysis of 39 RCTs by Tindall and colleagues showed significant improvements in fasting insulin and HOMA-IR in nut supplementation trials of median duration of 12 weeks. Perhaps surprisingly, however, there was no effect of supplementation with nuts on FPG compared to the low-nut control diet [24]. Subgroup analyses of baseline glycaemic status (normoglycaemia, prediabetes and T2D), body weight status (normal weight, overweight and obesity) or quantity of nuts consumed/day did not alter the main results [24]. The subgroup analyses conducted in our current study also failed to detect any differences between glycaemic (normoglycaemia–prediabetes) and ectopic fat (normal–high) subgroups within the main cohort. Individuals with prediabetes, and hence with higher baseline FPG and potential for abnormal pancreatic β-cell function, compared with those with normoglycaemia may be expected to be better responders to our current dietary intervention. This is similar for the high ectopic fat subgroup, where adverse effects of fat infiltration into the pancreas may be hypothesised to counteract any protective effects of the nut intervention on glycaemic endpoints. In our prior MRI-MRS study of pancreas and liver fat in individuals with overweight, we showed that the suppression of postprandial blood glucose achieved by the HP-NB product was countered in individuals with high organ fat content [30], confirming the role of ectopic fat deposition in moderating postprandial nutrient response.

Other key endpoints in our current intervention were fasting insulin and HOMA-IR, both previously reported to be altered by RCTs incorporating nuts into the diet [24]. However, there was no significant improvement in either fasting insulin concentration or the concurrent OGTT-induced insulin peak. The substitution of SFA and available CHO by unsaturated MUFA and PUFA may potentially contribute to an improvement of insulin sensitivity (i.e., HOMA-IR) and fasting insulin, although notably neither of these parameters were altered in our current trial. Perhaps the most likely explanation for the lack of protective effect of the nut product in our intervention compared with the previous literature is a dose–response effect. The median dose of nuts and peanuts in the Tindall meta-analysis was a high 52 g/day, with a range of 20–113 g/day, almost twice that of our HP-NB test product [24]. An intake of 28 g/day of mixed almonds and peanuts may have been insufficient to show an impact within 12 weeks. Unexpectedly, we observed an increase in HbA_1c_ in both diet groups during the intervention, for which there is also no clear explanation. Neither dietary product led to an adverse change in body weight or adiposity, nor was there significant worsening over time in any associated blood parameters in both diet groups. 

One concern for our current dietary intervention was the possibility that the nut bar would be included in the diet as an additional energy source, rather than as a substitution. Almonds and peanuts are high in energy, with the potential to increase body weight, and in turn worsen glycaemia. However, there was no evidence of significant changes in body weight or composition from either the ~1 MJ HP-NB or isoenergetic HC-CB groups, suggesting that participants adhered to the advice to substitute the intervention products for a meal or snack. Previous RCTs [12,19,53] which have substituted a nut-free diet with an isoenergetic almond- and pistachio-containing diet have reported similar positive findings. Similar outcomes were observed in an RCT investigating longer-term effects of the inclusion of walnuts in the diet [57]. A recent meta-analysis of RCTs also reported no significant effects of nut interventions on body weight, BMI, waist circumference or percentage body fat in T2D patients [58], supporting the inclusion of these products in the diet of individuals with overweight and dysglycaemia despite their high total fat content. 

Since edible nuts are present in diets in a great number of forms with variable compositions, the type of nut consumed is likely to be an important factor to consider. The 2019 meta-analysis by Tindall and colleagues [24] showed no significant effect of almonds [12,59,60,61,62,63,64], peanuts [65,66,67], cashew nuts [61,68] or walnuts [59,69,70] on FPG or HbA_1c_, but positive effects on HOMA-IR and fasting insulin. Only pistachio nuts, a tree nut of the cashew family, significantly decreased FPG [24] in addition to the markers of insulin sensitivity. However, perhaps unexpectedly, a recent 12-week RCT of pistachio nuts (57 g/day) consumed as a night-time snack in individuals with prediabetes [71] failed to show improvements in FPG, HbA_1c_, HOMA-IR or fasting insulin when compared with a positive control of ‘usual care’. We need to consider whether the lack of efficacy in our current study may have been due to the type of nut, almonds and peanuts, or the weight/dose of the nuts incorporated into the HP-NB diet. Insufficient intervention duration may be a further possible explanation. A prior meta-analysis reported greatest effects on glycaemia between nut-supplemented and control diets in studies of 12 week to 24 month durations [54]. However, conversely, the more recent Tindall meta-analysis failed to observe this effect in the sub-groups of long-term studies with duration > 12 weeks [24]. Tindall et al. also reported that no association was observed between the mean difference in FPG or other glycaemia-related outcomes and dose of nuts consumed.

There are some limitations to our study. Firstly, the Tū Ora RCT aimed to improve fasting glycaemia in participants with prediabetes at high risk of developing T2D. However, a cohort of individuals had reverted from dysglycaemia to normoglycaemia by the start of the study at CID1. This was unexpected and may have been due in part to a delay in the start of the intervention due to slower-than-predicted recruitment rates, or possibly changes in diet and lifestyle by participants after diagnosis of prediabetes at screening. Another possible explanation is the assessment of FPG at different laboratories at screening and CID1 timepoints. At both sites, blood samples collected at the screening visit were analysed daily in real time, from either whole blood (AKL) or plasma (WLG), whilst at CID1/baseline, FPG was batch analysed for both sites from frozen plasma samples. Both were validated analytical methods, and regularly calibrated within-site during the study. Finally, as glucose is a continuous biological variable, small natural variability within individuals may have moved them across the arbitrary threshold for the definition of prediabetes.

## 5. Conclusions

Our current intervention showed that an intake of ~28 g of nuts/day, consumed as a 1 MJ almond and peanut bar as a replacement for a meal or snack, over 12 weeks did not result in adverse weight gain or change in body composition in a cohort of Chinese adults with overweight. There was no suppression of FPG or related glycaemic endpoints, including fasting insulin and HOMA-IR. In subgroup analyses, there was no evidence that the presence of risk factors that promote susceptibility to T2D, including dysglycaemia and fat infiltration into the pancreas and liver, altered responses to the nut intervention, despite reporting these in our previous postprandial study [30]. A higher dose, longer intervention duration, and addition of other nut types, such as pistachio, may promote better responses to this nut intervention. 

## Figures and Tables

**Table 1:** Energy and macronutrient composition of the intervention products.

	HP-NB			HC-CB		
	g	kJ	%en	g	kJ	%en
One bar/day	50	1009	-	64	985	-
Total CHO	23	270	27	40	647	64
available CHO	12	205	20	38	635	63
starch	7	119	12	28	475	47
sugars	5	87	9	10	160	16
fibre	10	65	6	2	12	1
Total protein	8	130	13	4	74	7
Total fat	16	608	60	7	264	26
SFA	2	72	7	1	24	2
MUFA	9	333	34	4	148	15
PUFA	5	185	19	2	74	7
Nutrient Profile Score	−2			3		

CHO, carbohydrate; HP-NB, higher-protein nut bar; HC-CB, higher-carbohydrate cereal bar; SFA, saturated fatty acid; MUFA monounsaturated fatty acid; PUFA, polyunsaturated fatty acid. Calculated using FoodWorks^®^ (v8, Xyris Software, Melbourne, Australia). Nutrient Profile Score (NPS) calculated using the NPS Calculator [35]. For Category 2 (solid food), NPS < 4 meets nutrient profiling scoring criterion (NPSC) for Australia and New Zealand as suitable for a health claim.

**Table 2:** Participant characteristics at baseline for all and sub-group diet, glycaemia, ectopic fat.

	All	Diet			Glycaemia			^5^ Ectopic Fat		
		HP-NB	HC-CB	*p*	Normo- Glycaemia	Pre- Diabetes	*p*	NEF	HEF	*p*
n	101	53	48		61	40		36	63	
Age (y)	46.3, 11.1	46.6, 10.4	45.9, 12.0	0.750	44.9, 10.8	48.5, 11.4	0.106	44.2, 11.4	47.7, 10.7	0.127
Body weight (kg)	76.3, 14.8	77.6, 16	74.7, 13.3	0.323	74.5, 13.8	79.0, 16.0	0.138	70.8, 10.3	79.6, 16.2	**0.004**
BMI (kg/m^2^)	27.4, 3.6	27.6, 3.7	27.2, 3.5	0.577	27.0, 3.1	28.1, 4.2	0.146	26.0, 2.6	28.3, 3.9	**0.003**
Waist circumf (cm)	91.7, 10.8	92.4, 11.2	90.9, 10.4	0.482	89.2, 9.1	95.5, 12.1	**0.004**	86.5, 8.5	94.7, 11.0	**<0.0001**
Hip circumf (cm)	102.7, 8.0	103.2, 8.8	102.0, 7.0	0.453	102.3, 6.9	103.2, 9.4	0.581	100.3, 6.7	103.9, 8.5	**0.031**
SBP (mmHg)	117, 16	119, 17	116, 15	0.316	116, 16	119, 16	0.407	109, 15	122, 14	**<0.0001**
DBP (mmHg)	72, 13	75, 13	69, 11	**0.027**	71, 13	74, 12	0.216	68, 10	75, 13	**0.008**
*Glycaemic markers*										
^1^ Screen FPG (mmol/L)	6.1, 0.4	6.1, 0.4	6.1, 0.4	0.473	6.0, 0.3	6.2, 0.4	**0.004**	6.1, 0.4	6.1, 0.3	0.366
FPG (mmol/L)	5.5, 0.6	5.6, 0.6	5.5, 0.5	0.838	5.2, 0.3	6.1, 0.5	**<0.0001**	5.4, 0.6	5.6, 0.5	0.206
HbA_1c_ (mmol/mol)	36.3, 10.5	36.1, 11.0	36.4, 10.1	0.902	32.7, 6.9	41.7, 12.6	**<0.0001**	35.3, 10.9	36.5, 10.3	0.601
Insulin (uU/mL)	13.2, 9.3	12.4, 8.4	14.1, 10.2	0.378	10.2, 6.9	17.9, 10.5	**<0.0001**	10.3, 5.3	15.1, 10.6	**0.013**
HOMA-IR	3.4, 2.6	3.2, 2.3	3.6, 2.8	0.443	2.4, 1.7	4.9, 3.0	**<0.0001**	2.5, 1.4	3.9, 3.0	**0.013**
*Lipid markers*										
Total chol (mmol/L)	4.9, 0.9	5.1, 0.7	4.7, 0.9	**0.013**	4.7, 0.8	5.2, 0.8	**0.003**	5.0, 0.9	4.9, 0.8	0.678
LDL-C (mmol/L)	3.0, 0.7	3.2, 0.7	2.9, 0.7	**0.029**	2.9, 0.7	3.3, 0.7	**0.017**	3.0, 0.8	3.1, 0.7	0.907
HDL-C (mmol/L)	1.2, 0.3	1.2, 0.3	1.2, 0.3	0.963	1.2, 0.3	1.1, 0.3	0.064	1.3, 0.3	1.1, 0.3	**0.038**
TG (mmol/L)	1.6, 0.9	1.6, 0.9	1.5, 1	0.436	1.4, 0.7	1.9, 1.2	**0.006**	1.5, 1.1	1.6, 0.8	0.348
*Inflammatory markers*										
hsCRP (mg/L)	13.3, 24.4	12.5, 23.1	14.1, 25.9	0.725	15.5, 30.0	9.9, 11.7	0.264	9.8, 22.7	15.6, 25.5	0.258
BDNF (ng/mL)	836, 601	855, 716	815, 449	0.735	940, 665	678, 453	**0.031**	782, 428	862, 690	0.527
DKK-1 (ng/mL)	17.1, 18.3	19.5, 24.0	14.5, 8.3	0.175	15.3, 8.9	19.8, 26.9	0.237	19.5, 27.3	16.0, 10.5	0.353
IL-6 (pg/mL)	3.6, 5.2	4.2, 6.9	2.8, 1.8	0.177	2.9, 2.3	4.6, 7.7	0.116	3.4, 3.7	3.7, 5.9	0.843
S100beta (pg/mL)	216, 163	230, 161	198, 165	0.352	199, 144	241, 187	0.218	226, 167	212, 163	0.700
^2^ *DXA body composition*										
Total FM (kg)	25.8, 6.8	26.2, 6.8	25.3, 6.8	0.493	25.7, 6.1	25.8, 7.8	0.933	23.6, 5.1	27.1, 7.4	**0.014**
Total FFM (kg)	50.5, 10.8	51.3, 11.3	49.5, 10.2	0.412	48.8, 10.6	52.9, 10.8	0.072	47.1, 9.1	52.7, 11.3	**0.015**
Total FM (%)	33.7, 5.9	33.9, 5.3	33.5, 6.5	0.501	34.6, 5.8	32.4, 5.8	0.256	33.4, 5.6	33.8, 6.1	0.733
AbFM (%)	40.6, 6.8	41.3, 6.1	39.9, 7.5	0.154	41.1, 7.3	39.9, 6.1	0.781	37.9, 7.1	42.1, 6.3	**0.003**
*MRI/MRS imaging*										
VAT:SAT ratio	0.66, 0.29	0.69, 0.24	0.63, 0.33	0.328	0.58, 0.23	0.78, 0.31	**0.002**	0.55, 0.24	0.73, 0.29	**0.001**
^3^ Pancreas fat (%)	4.8, 1.7	5.2, 1.7	4.4, 1.6	**0.029**	4.5, 1.6	5.3, 1.7	**0.031**	3.3, 0.8	5.7, 1.4	**<0.0001**
^4^ Liver fat (%)	5.2, 5.8	4.5, 4.4	6.0, 7.0	0.242	4.4, 5.1	6.4, 6.6	0.089	2.2, 1.6	7.0, 6.6	**<0.0001**

Mean and SD. *p* < 0.05 pairwise comparisons shown in bold type. AbFM, abdominal fat mass; BDNF, brain-derived neurotrophic factor; BMI, body mass index; DBP, diastolic blood pressure; DKK-1, Dickkopf-1; DXA, dual-energy X-ray absorptiometry; FFM, fat-free mass; FM, fat mass; FPG, fasting plasma glucose; HC-CB, higher-CHO cereal bar; HDL-C, high-density lipoprotein cholesterol; HEF, high ectopic fat—pancreas and/or liver; HOMA-IR, homeostatic model assessment of insulin resistance; HP-NB, higher-protein nut bar; hsCRP, high-sensitivity C-reactive protein; IL-6, Interleukin-6; LDL-C, low-density lipoprotein cholesterol; NEF, normal ectopic fat—pancreas and/or liver; SAT, subcutaneous adipose tissue; SBP, systolic blood pressure; S100beta, S100 calcium-binding protein beta; TG, triglyceride; and VAT, visceral adipose tissue. ^1^ FPG calculated at screen visit as fasting venous glucose (mmol/L) × 1.11 [51], AKL site; ^2^ DXA, n = 96: ^3^ pancreas fat, n = 98; ^4^ liver fat, n = 100; and ^5^ ectopic fat, all n = 99.

**Table 3:** Compliance over the 12-week intervention.

	HP-NB		HC-CB	
	Allocated to Diet Group	Completers	Allocated to Diet Group	Completers
n	53	51	48	46
Total bars consumed	58, 14	59, 12	60, 11	62, 10
Compliance (%)	80, 19	82, 17	84, 16	85, 13

Mean and SD. 100% compliance = 72 bars (6 bars × 12 weeks). HP-NB, higher-protein nut bar; HC-CB, higher-carbohydrate cereal bar.

**Table 4:** Anthropometry and clinical markers at baseline and 12 weeks.

Variables	HP-NB		HC-CB		*p*		
	Baseline	12 Weeks	Baseline	12 Weeks	Time	Diet	Diet*Time
n	53	51	48	46			
Body weight (kg)	77.6, 2.2	77.0, 2.3	74.7, 1.9	74.8, 2.1	0.999	**0.054**	0.999
Body mass index, BMI (kg/m^2^)	27.6, 0.5	27.3, 0.6	27.2, 0.5	27.3, 0.6	0.998	0.409	0.995
Waist circumference (cm)	92.4, 1.5	91.7, 1.7	90.9, 1.5	89.9, 1	0.578	0.269	0.911
Hip circumference (cm)	103.2, 1.2	101.6, 1.2	102.0, 1	101.8, 1.2	0.444	0.671	0.552
SBP (mmHg)	119, 2	117, 2	116, 2	116, 2	0.710	0.429	0.485
DBP (mmHg)	75, 2	68, 2	69, 2	65, 1	**0.002**	**0.011**	0.392
*Fasting clinical markers*							
FPG (mmol/L)	5.6, 0.1	5.5, 0.1	5.5, 0.1	5.5, 0.1	0.509	0.822	0.991
HbA_1c_	36.1, 1.5	40.2, 1.3	36.4, 1.6	39.5, 1.4	**0.016**	0.865	0.725
Insulin (µIU/mL)	12.4, 1.1	15.3, 1.8	14.1, 1.5	14.8, 2.3	0.690	0.359	0.949
HOMA-IR	3.2, 0.3	3.8, 0.5	3.6, 0.4	3.8, 0.7	0.711	0.350	0.991
Total cholesterol (mmol/L)	5.1, 0.1	5.1, 0.1	4.7, 0.1	4.8, 0.1	0.803	**0.002**	0.835
LDL-C (mmol/L)	3.2, 0.1	3.2, 0.1	2.9, 0.1	2.9, 0.1	0.866	**0.017**	0.741
HDL-C (mmol/L)	1.2, 0.0	1.2, 0.0	1.2, 0.1	1.2, 0.1	0.573	0.694	0.648
TG (mmol/L)	1.6, 0.1	2.0, 0.3	1.5, 0.1	1.5, 0.1	0.248	0.095	0.359
hsCRP (mg/L)	12.5, 3.1	7.0, 1.3	14.1, 3.7	6.4, 1.3	**0.014**	0.844	0.654
BDNF (ng/mL)	855, 98	741, 72	815, 65	666, 68	0.080	0.507	0.887
DKK-1 (ng/mL)	19.5, 3.3	19.6, 3.3	14.5, 1.2	13.2, 1.2	0.830	**0.027**	0.763
IL-6 (pg/mL)	4.2, 1.3	4.5, 1.1	2.8, 0.3	2.6, 0.3	0.786	**0.037**	0.795
S100beta (pg/mL)	230, 26	220, 25	198, 27	168, 18	0.294	0.095	0.779
*OGTT-glucose markers*							
ΔC_max_ (mmol/L)	4.7, 0.3	5.2, 0.4	4.6, 0.3	4.7, 0.3	0.431	0.481	0.651
iAUC_glucose 0–120min_ (mmol/l*min)	348, 30	385, 33	330, 29	357, 31	0.299	0.465	0.870
*OGTT-insulin markers*							
ΔC_max_ (µIU/mL)	154.9, 13.3	156, 12.7	148.5, 11.8	159.9, 13.4	0.551	0.934	0.556
iAUC_insulin 0–120min_ (µIU/mL*min)	10,942, 920	10,991, 894	10,134, 854	11,393, 1010	0.508	0.820	0.518

Mean and SEM. *p* < 0.05 shown in bold type. BDNF, brain-derived neurotrophic factor; BMI, body mass index; DBP, diastolic blood pressure; DKK-1, Dickkopf-1; FPG, fasting plasma glucose; HbA_1c_, haemoglobin A_1c_; HDL-C, high-density lipoprotein cholesterol; HC-CB, higher-CHO cereal bar; HOMA-IR, homeostatic model assessment of insulin resistance; HP-NB, higher-protein nut bar; hsCRP, high-sensitivity C-reactive protein; IL-6, interleukin-6; LDL-C, low-density lipoprotein cholesterol; SBP, systolic blood pressure; S100beta, S100 calcium-binding protein beta; TG, triglyceride; OGTT, oral glucose tolerance test; ΔC_max_, peak concentration; iAUC_glucose 0–120min_, incremental area under the curve glucose, 120 min; and iAUC_insulin 0–120min_, incremental area under the curve insulin, 120 min.

## Data Availability

De-identified data may be shared and made available upon reasonable request to the corresponding author and subject to an approved proposal and data access agreement due to ethical reasons.
